# Factors Associated with Successful Completion of the Chronic Disease Self-Management Program among Middle-Aged and Older Asian-American Participants: A National Study

**DOI:** 10.3389/fpubh.2014.00257

**Published:** 2015-04-27

**Authors:** SangNam Ahn, Matthew Lee Smith, Jinmyoung Cho, Luohua Jiang, Lindsey Post, Marcia G. Ory

**Affiliations:** ^1^Division of Health Systems Management and Policy, School of Public Health, The University of Memphis, Memphis, TN, USA; ^2^Department of Health Promotion and Community Health Sciences, School of Public Health, Texas A&M Health Science Center, College Station, TX, USA; ^3^Department of Health Promotion and Behavior, College of Public Health, The University of Georgia, Athens, GA, USA; ^4^Center for Applied Health Research, Baylor Scott & White Healthcare, Temple, TX, USA; ^5^Department of Epidemiology, School of Medicine, University of California Irvine, Irvine, CA, USA

**Keywords:** Asian-Americans, chronic disease management, Chronic Disease Self-Management Program, evidence-based programs

## Abstract

Asian-Americans are a small but fast-growing population in the United States who are increasingly experiencing multiple chronic diseases. While the evidence-based Chronic Disease Self-Management Program (CDSMP) has been disseminated among various racial and ethnic populations, few studies specifically investigate participants with an Asian background. The study aims to identify characteristics of middle-aged and older Asian-American CDSMP participants (older than 50 years) and investigate factors related to successful workshop completion (i.e., attending 4+ of the 6 sessions) among this population. Data were analyzed from 2,716 middle-aged and older Asian-Americans collected during a 2-year national dissemination of CDSMP. Multilevel logistic regression analyses were conducted to identify individual- and workshop-level covariates related to successful workshop completion. The majority of participants were female, living with others, and living in metro areas. The average age was 71.3 years old (±9.2), and the average number of chronic conditions was 2.0 (±1.5). Successful completion of CDSMP workshops among participants was associated with their number of chronic conditions (OR = 1.10, *P* = 0.011), living in non-metro areas (OR = 1.77, *P* = 0.009), attending workshops from area agencies on aging (OR = 1.56, *P* = 0.018), and attending a workshop with higher completion rates (OR = 1.03, *P* < 0.001). This study is the first large-scale examination of Asian-American participants enrolled in CDSMP and highlights characteristics related to intervention attendance among this under-studied minority population. Knowing such characteristics is important for serving the growing number of Asian-Americans with chronic conditions.

## Introduction

Almost three-quarters of Asian-American adults are foreign-born, representing many countries of origin, including China, India, and the Philippines (1). Collectively, these subgroups constitute the fastest-growing ethnic group in the country, representing almost 6% of the U.S. population (1). The Asian-American population in the U.S., which was estimated at 18.9 million in 2012 (2), grew by 46% from 2000 to 2010, and is outpacing the growth of other racial/ethnic groups. Between 2011 and 2012, the rate of population increase was 2.9% for Asian-Americans, 2.2% for Hispanics; 2.2% for Native Hawaiians and Other Pacific Islanders; 1.5% for American-Indians and Alaska Native; and 1.3% for African-Americans (2). This population growth warrants further study of health conditions among Asian-Americans. Although the prevalence rate of chronic conditions among Asian-Americans (42%) is lower than their African-American (77%), Latino (68%), and White (64%) counterparts, the burden of chronic conditions among Asian-Americans should be carefully scrutinized based on population projections (2–4). As the total population of Asian-Americans increases, it is expected that a greater number of Asian-Americans will suffer from chronic conditions.

The Chronic Disease Self-Management Program (CDSMP) has been introduced and widely disseminated in the U.S. as a method to empower patients with self-management skills to deal with their chronic conditions (5). Drawing upon social learning theory (6), CDSMP is an evidence-based, peer-led intervention consisting of six highly participative classes held for 2.5 h each, once a week, for six consecutive weeks (5). CDSMP has resulted in improved healthcare and health (7, 8), while potentially saving healthcare costs (9). While CDSMP has been successfully disseminated among diverse populations, there are few studies focusing specifically on the characteristics of middle-aged or older Asian-Americans enrolled in CDSMP or examining the factors associated with completing CDSMP in this population. Previous studies have shown that Asian-Americans complete CDSMP at a somewhat higher rate than the general participant population and at about the same rate as White participants (10). Thus, the objective of the study was to analyze the dataset more closely to (1) identify characteristics of Asian-American CDSMP participants in the 2010–2012 national dissemination of CDSMP in the U.S.; and (2) identify the factors associated with CDSMP completion among middle-aged and older Asian-American participants.

## Methods

### Data source and study population

Cross-sectional data for this study were retrospectively obtained from a nationwide delivery of CDSMP as part of the American Recovery and Reinvestment Act of 2009 (i.e., Recovery Act) *Communities Putting Prevention to Work: CDSMP initiative* (11). The U.S. Administration on Aging led this initiative in collaboration with the Centers for Disease Control and Prevention and the Centers for Medicare and Medicaid Services to support the translation of CDSMP in 45 states, Puerto Rico, and the District of Columbia (12). This initiative was conducted between 2010 and 2012 with the goal of reaching the diverse population of the Americans embedding the delivery structures into statewide systems (11). Within the first 2 years of this initiative, there were more than 100,000 adults participating in 9,305 workshops in 1,234 U.S. counties (11). For this study, data were analyzed from 2,716 Asian-American participants (i.e., aggregate Asian ethnic groups) who aged 50 years or older and responded to all relevant survey questions.

## Measures

### Dependent variable

Chronic Disease Self-Management Program workshop attendance was the dependent variable for this study. Successful completion was defined as attendance at four or more of the six workshop sessions, which is consistent with definitions used by the program developers and in a variety of other studies (7–9, 11).

### Individual- or neighborhood-level covariates

As individual-level covariates, socio-demographic factors included age (in years), sex (male vs. female), and living arrangement (living with others vs. living alone). Health status was measured by the number of self-reported chronic conditions (i.e., arthritis, cancer, depression, diabetes, heart disease, hypertension, lung disease, stroke, osteoporosis, and other chronic conditions). As neighborhood-level covariates, median household income (in $10,000 units) was included based on the participants’ ZIP Code. Rural–Urban Commuting Area Codes based on participants’ ZIP Code information were used to categorize participants’ residence (metro vs. non-metro) (13).

### Workshop-level covariates

Workshop delivery sites included area agencies on aging (AAA)/senior centers, healthcare organizations, residential facilities, community or multi-purpose centers, faith-based organizations, educational institutions, recreational centers, tribal centers, and workplaces. The last four delivery sites made up <13% of the total and were coded as “other” for purpose of the study and their low distribution (<13%). Workshop composition varied in the proportion of Asian-Americans participating. We hypothesized that workshops with more racial/ethnic homogeneity might have higher completion rates due to shared culture and language (14, 15). Workshops with larger proportions of participants successfully completing the intervention might also signify greater social cohesion and support (i.e., higher completion workshop). We also hypothesized that Asian-American participants in workshops with higher overall completion rates would have higher completion rates themselves (16). As such, we computed the percentages of Asian-Americans and successful completers in each workshop. To avoid endogeneity issue, we excluded the current participant from their workshop when calculating the workshop completion rate. In other words, the resulting workshop completion rate represents the average completion rate among the classmates of each participant. The proportions of Asian-American participants and average workshop completion rates were included in analyses as workshop-level covariates.

### Statistical analysis

To compare the characteristics of the participants who completed the CDSMP workshop to those who did not, we used χ^2^-tests for categorical independent variables and two-sample *t*-test for continuous independent variables. Multilevel logistic regression models were used to investigate the association between successful workshop completion and individual-level, as well as workshop-level covariates. First, all individual-level independent variables were introduced into a multilevel logistic regression model (Model 1). Then, we generated another multilevel logistic regression model after including workshop-level variables (Model 2). The proportion of variance explained (PVE) at the workshop level by different levels of variables was calculated as: PVE = (*V*_0_ − *V*_1_)/*V*_0_ × 100, where *V*_0_ is the second-level variance of the Null Model, and *V*_1_ is the second-level variance of the adjusted model (17). Multilevel analyses were conducted using the “gllamm” command in Stata 11 (18).

## Results

Table 1 shows frequency distributions of independent variables among the total population, and then divided by workshop completion status; 79.5% of participants successfully completed CDSMP workshops (*n* = 2,159) and 20.5% did not (*n* = 557). As a whole, Asian-American participants were predominantly female (73.1%), living with others (97.5%), and living in metro areas (91.3%). The average age was 71.3 years old (±9.2), and the average number of chronic conditions was 2.0 (±1.5).

**Table 1:** **Characteristics of middle-aged and older Asian-American Chronic Disease Self-Management Program participants by CDSMP completion (*N* = 2,716)**.

	Total (*N* = 2,716)	CDSMP non-completion (*N* = 557)	CDSMP completion (*N* = 2,159)	
	*N* (%)	*N* (%)	*N* (%)	*P-*value*
Female	1,985 (73.1)	397 (71.3)	1, 588 (73.6)	0.280
Living alone	68 (2.5)	19 (3.4)	49 (2.3)	0.124
Rural–urban commuting area codes				0.009
Metro	2,479 (91.3)	524 (94.1)	1, 955 (90.6)	
Non-metro	237 (8.7)	33 (5.9)	204 (9.5)	
Workshop delivery site				0.010
Other	341 (12.6)	77 (13.8)	264 (12.2)	
Area agency on aging/senior centers	741 (27.3)	123 (22.1)	618 (28.6)	
Health care organization	379 (14.0)	77 (13.8)	302 (14.0)	
Residential facilities	431 (15.9)	111 (19.9)	320 (14.8)	
Community/multi-purpose	720 (26.5)	148 (26.6)	572 (26.5)	
Faith-based organization	104 (3.8)	21 (3.8)	83 (3.8)	

	**Mean (±SD)**	**Mean (±SD)**	**Mean (±SD)**	***P*-value^a^**

Age in years (from 50 to 101)	71.3 (±9.2)	71.2 (±9.5)	71.4 (± 9.2)	0.670
Neighborhood median income in $10,000 unit (from 2.2 to 11.6)	6.2 (±1.4)	6.2 (±1.5)	6.2 (± 1.3)	0.466
Number of chronic conditions (from 0 to 10)	2.0 (±1.5)	1.9 (±1.4)	2.1 (± 1.5)	0.009
% Of Asian-Americans in the workshop	57.5 (±36.8)	53.2 (±36.7)	58.6 (± 36.7)	0.002
Workshop completion rate (%)	74.9 (±18.7)	66.4 (±18.7)	77.1 (± 18.0)	<0.001

*P-value for χ^2^-test comparing the participants who completed the CDSMP workshop and who did not. Other workshop delivery sites include educational institutions, recreational centers, tribal centers, and workplaces.

*^a^*P-*value for two-sample *t*-test comparing the participants who completed the CDSMP workshop and who did not*.

Chronic Disease Self-Management Program completion was not significantly different in terms of age, sex, neighborhoods median income, or living arrangement. Considering individual-level variables, participants living in non-metro areas had significantly higher completion rates than their urban counterparts (*P* = 0.009). The number of chronic conditions was higher among those who completed CDSMP workshops relative to those who did not (*P* = 0.009). All workshop-level variables significantly differentiated between those who completed CDSMP workshops and who did not. Those who attended AAA delivery sites were more likely to complete CDSMP workshops, whereas those who attended residential facilities or other sites had lower completion rates (*P* = 0.010). In addition, CDSMP workshop completion was positively associated with the percentage of Asian-American participants (*P* = 0.002) and the workshop completion rate in each workshop (*P* < 0.001).

Table 2 shows results of the multilevel logistic regressions including the Null Model (i.e., intercept-only model), Model 1 (only including individual-level variables), and Model 2 (including both individual-level and workshop-level variables). In Model 1, a higher number of chronic conditions and living in non-metro areas increased the odds of completing CDSMP workshops (OR = 1.09, *P* = 0.030; OR = 1.84, *P* = 0.025, respectively). In Model 2, the odds of completing CDSMP workshops increased among participants with a higher number of chronic conditions (OR = 1.10, *P* = 0.011), living in non-metro areas (OR = 1.77, *P* = 0.009), and those who attended a workshop with a higher completion rate (OR = 1.03, *P* < 0.001). Those who attended workshops from AAA (OR = 1.56, *P* = 0.018) were more likely to complete CDSMP workshops compared to those who attended CDSMP workshops from other delivery sites (i.e., educational institution, recreational center, tribal center, and workplace). The second-level variance explained by the individual-level variables (in Model 1) was (1.45–1.41)/1.45 × 100 = 2.8%, indicating that the individual-level variables explained 2.8% of the variability found at the second-level compared with the Null Model. Meanwhile, the second-level variance explained by the workshop-level variables (in Model 2) was (1.41–0.16)/1.41 × 100 = 88.7%, indicating that the workshop-level variables explained 88.7% of the variability found at the second-level compared with the Model 1.

**Table 2:** **Individual and workshop characteristics associated with successful completion of Chronic Disease Self-Management Program (CDSMP) among middle-aged and older Asian-American participants (*N* = 2,716)**.

	Empty model	Model 1	Model 2
	Adjusted OR (95% CI)	Adjusted OR (95% CI)	Adjusted OR (95% CI)
**Individual characteristics**
Age	–	1.00 (0.99–1.01)	1.00 (0.99–1.02)
Sex
Male	–	Ref.	Ref.
Female	–	1.12 (0.88–1.43)	1.08 (0.86–1.35)
Number of chronic conditions	–	**1.09 (1.01**–**1.18)**	**1.10 (1.02**–**1.18)**
Neighborhood median income	–	1.06 (0.96–1.18)	1.04 (0.96–1.12)
Living arrangement
Living with others	–	Ref.	Ref.
Living alone	–	0.69 (0.36–1.31)	0.71 (0.40–1.27)
Rural–urban commuting area codes
Metro	–	Ref.	Ref.
Non-metro	–	**1.84 (1.08**–**3.15)**	**1.77 (1.15**–**2.71)**
**Workshop characteristics**
Workshop delivery site
Other	–	–	Ref.
Area agency on aging/senior centers	–	–	**1.56 (1.08**–**2.25)**
Health care organization	–	–	1.42 (0.94–2.15)
Residential facilities	–	–	1.12 (0.76–1.67)
Community/multi-purpose	–	–	1.35 (0.91–2.00)
Faith-based organization	–	–	1.21 (0.64–2.27)
% Of Asian-Americans in the workshop	–	–	1.00 (0.99–1.01)
Workshop completion rate (%)	–	–	**1.03 (1.02**–**1.03)**
Between-area variation (SE)	1.45 (0.30)	1.41 (0.30)	0.16 (0.14)

*Other workshop delivery sites include educational institutions, recreational centers, tribal centers, and workplaces*.

*OR are printed in bold if *P* < 0.05*.

*OR, odds ratio; 95% CI, 95% confidence interval; Ref., referent*.

## Discussion

This retrospective analysis is unique in that it identifies correlates significantly related to CDSMP completion at individual- and workshop-levels among Asian-American participants. The study findings are especially relevant given that Asian-Americans are the fastest-growing population in the U.S. The current study reveals that Asian-American participants are similar to other CDSMP participants in this national dissemination in terms of being predominantly female, living with others, residing in metro areas, and having multiple chronic conditions (7, 11). Workshop completion was positively associated with number of chronic conditions, attending workshops from AAA, attending higher completion workshops, and rural residence. These factors independently or in combination contributed to the 80% CDSMP completion rate among Asian-American participants. Recent studies utilizing the same dataset reported that the average CDSMP completion rate was 75% (*n* = 89,861) (10, 11), which is slightly lower than that (i.e., 80%) of the Asian-American participants shown in the current study. They also found no significant difference in completion rates between Asian-American and White participants (10). However, there has been no further analysis of the factors that might influence completion rates among Asian-Americans.

The positive association between the participants’ health status (i.e., represented by the number of chronic conditions) and their likelihood of completing the program is consistent with recent CDSMP evaluations (8). This result is relatively encouraging given that an earlier study found those with chronic conditions had lower attendance rates of behavioral interventions in the community (19). Mobility issues related to chronic conditions have been cited as probable barriers to intervention adherence (20, 21). There is little evidence to help us disentangle the positive association between severe health conditions and workshop completion. However, when study participants engage in highly participative workshops focusing on goal setting and problem solving skills needed by those coping with multiple chronic conditions, their motivations to attend these workshops may outweigh other factors, which would limit attendance, such as pain, depression, or mobility issues. As such, this research highlights the importance of recognizing the value of prevention across the disease continuum and especially targeting those with multiple chronic conditions for CDSMP.

Rural residence seems to be an important factor related to CDSMP completion among Asian-Americans. The current study reveals 8.7% of participants reported living in non-metro areas, which is two times greater than the 2010 Census estimates of rural-residing Asian-Americans (4.3%) (22). In the current study including both individual- and workshop-level variables, Asian-American participants who lived in non-metro areas were 77% more likely to complete CDSMP compared to those who lived in metro areas. There are multiple potential explanations for this finding. First, there could be more social integration in rural communities so that participants could be more likely to know leaders, organizers, and participants, leading to a natural support system for encouraging attendance. Similarly, it could be easier to “get the word out” to participants in rural communities once rural informants were reached. Alternatively, if fewer participants are enrolled in rural workshops, it might be easier for leaders to provide reminders about workshop sessions and communicate with participants outside of workshop time. In our additional analysis, average number of participants enrolled in rural workshops (i.e., 11.7) was significantly lower than that of urban workshops (i.e., 14.2) (*P* < 0.001). Second, the general lack of access to healthcare in rural areas (23, 24) might encourage participants in rural areas to take advantage of available behavioral interventions like CDSMP. More in-depth analysis is needed to reveal which aspects of the non-metro CDSMP facilitated more successful completion. Along this line, future studies should further investigate the extent to which rural residence affects completion of evidence-based programs among Asian and other smaller racial/ethnic groups.

When workshop-level variables were included in the analysis, one of the interesting results was the significant association between program completion and specific delivery site. Asian-American CDSMP participants were more likely to complete CDSMP when they attended workshops from AAA. In a sense, AAAs or senior centers are especially advantaged in delivering evidence-based programs to minority groups because AAAs have had a longer experience of providing educational services or congregate meals that have attracted diverse populations (25). This early advantage is strengthened by new mandates for AAAs to deliver evidence-based programs for diverse groups of seniors (25). Nevertheless, the association between delivery sites and CDSMP completion requires another look while considering participants’ residence (metro vs. non-metro). In an additional analysis, we found that AAA (28.2%) and community/multi-purpose centers (28.8%) were the two most common delivery sites in metro areas, whereas other (i.e., educational institution, recreational center, tribal center, and workplace) (35.2%) and residential facilities (19.0%) were two most common delivery sites in non-metro areas. This may indicate that adding the workshop characteristics in Model 2 does not seem to confound the relationship between rural residence and workshop completion anymore when looking at the relatively small changes of odds ratios of rural residence covariate (from 1.84 in Model 1 to 1.77 in Model 2). Nevertheless, these results may require additional study to address unanswered questions: which delivery sites can reach out to diverse populations? What factors related to delivery sites might be associated with more successful completion of CDSMP (e.g., rural residence or specific delivery sites or both)?

As part of the workshop-level variables, Asian-American participants who attended workshops with higher completion rates were more likely to complete the program. There are very few studies explaining variation of completing interventions in terms of percentage of intervention completers among specific racial and ethnic groups. One of the plausible explanations would be related to efficient or engaging CDSMP leaders. These leaders may instruct workshops in a way that provides participants with more enjoyment or educational benefit from each session, thereby increasing participants’ motivation to complete the program. Alternatively, when a large portion of participants in a workshop were diligently engaging in each session, other “less-interested” participants may have been effectively encouraged to complete the program through a form a positive peer pressure. These factors independently or together, coupled with an assumption that Asian people feel more comfortable in a group (i.e., collectivism rather than individualism) and tend to follow the group (26) might boost attendance and increase complete rates of CDSMP. While it was assumed that Asian-American participants in classes with higher proportions of Asian-Americans would be more likely to complete CDSMP, this *a priori* hypothesis was not supported. Moreover, the current study also found that utilizing the multilevel analysis (i.e., individual- and workshop-levels) is highly recommended since including workshop-level variables explained more than 88% of the second-level variance.

Despite the study’s unique contribution to the literature, some limitations should be considered. First, these results were based on cross-sectional data, which limit our ability to determine a causal relationship between any of the variables and CDSMP completion. Second, the study participants are not nationally representative, which limits the generalizability of these study findings. Third, our data relied on program participants’ self-reported measurements that may generate recall bias or social desirability bias. However, we were not able to find problematic patterns related to this concern. Third, and most importantly, it is over-simplistic to lump all Asian-Americans together in one category. Because of the multiplicity of nationalities and unique cultures in Asia, it is difficult to make generalizations or draw conclusions about such a broadly defined, diverse population. Additionally, we did not include measures, which would clarify participants’ level of acculturation that likely influence the variables’ effects on program participation or workshop completion. Unfortunately, the available data did not allow distinctions among different Asian-Americans; however, we recommend that sub-classifications of Asian-American participants be collected and analyzed in future studies. Nevertheless, the primary purpose of this study was to explore the characteristics of Asian-American CDSMP participants and contributing factors related to program completion, and as such offer initial insights that can be explored further.

## Conclusion

The underlying value of our study is the potential to improve the implementation and dissemination of successful evidence-based programs among Asian-Americans. A major study conclusion is that completion rates among Asian-American CDSMP participants were high (approximately 80% of participants), but they could be improved with careful targeting of these populations based on health status, participant’s residence, and workshop delivery sites. In this way, our findings can inform policy makers, program coordinators, and workers in the field who want to expand CDSMP utilization among Asian-Americans. The crucial next step will be focusing on improving implementation and dissemination of CDSMP among diverse segments of Asian-Americans. Such actions can help the growing population of Asian-Americans achieve improvements in health and health care outcomes.

## Conflict of Interest Statement

The authors declare that the research was conducted in the absence of any commercial or financial relationships that could be construed as a potential conflict of interest.

This paper is included in the Research Topic, “Evidence-Based Programming for Older Adults.” This Research Topic received partial funding from multiple government and private organizations/agencies; however, the views, findings, and conclusions in these articles are those of the authors and do not necessarily represent the official position of these organizations/agencies. All papers published in the Research Topic received peer review from members of the Frontiers in Public Health (Public Health Education and Promotion section) panel of Review Editors. Because this Research Topic represents work closely associated with a nationwide evidence-based movement in the US, many of the authors and/or Review Editors may have worked together previously in some fashion. Review Editors were purposively selected based on their expertise with evaluation and/or evidence-based programming for older adults. Review Editors were independent of named authors on any given article published in this volume.
